# Ileitis Secondary to Oral Capecitabine Treatment?

**DOI:** 10.1155/2012/154981

**Published:** 2012-11-26

**Authors:** Rami Radwan, Wanangwa C. Namelo, Mark Robinson, Alison E. Brewster, Gethin L. Williams

**Affiliations:** ^1^Department of Colorectal Surgery, Royal Gwent Hospital, Newport NP20 2UB, UK; ^2^Department of Clinical Oncology, Velindre Hospital, Cardiff CF14 2TL, UK; ^3^Department of Radiology, Royal Gwent Hospital, Newport NP20 2UB, UK

## Abstract

The efficacy of capecitabine as adjuvant therapy in colon cancer is well demonstrated and its lower toxicity rates when compared with 5-FU make it an increasingly more favourable option for patients. This case highlights the awareness of a potentially severe side effect related to the use of capecitabine, yet through the early identification of symptoms patients can be managed conservatively.

## 1. Introduction

Colon Cancer remains one of the most common cancers worldwide and a leading cause of death in the western world [1]. Capecitabine is an oral fluoropyrimidine used as adjuvant chemotherapy in both stage III, and recently, stage II colon cancer. Small bowel toxicity following administration of intravenous chemotherapeutic agents is uncommon, and to date, similar side effects in patients treated solely with oral capecitabine have not been observed. In this paper, we present an unusual case of ileitis following administration of oral capecitabine. 

## 2. Case Report

A 67-year-old gentleman with no significant comorbidities presented to our emergency department with a two-day history of worsening abdominal pain and distension. Abdominal X-ray demonstrated large bowel obstruction and a subsequent CT abdomen revealed an obstructing midtransverse colon tumour with no obvious distant metastases. He underwent an emergency right hemicolectomy with a primary anastomosis. Postoperative histology showed a 5 cm moderately differentiated adenocarcinoma (Dukes B; T4/N0). Immunohistochemistry identified microsatellite stability (MSS) and it was felt he would benefit from adjuvant chemotherapy.

**Figure 1:**
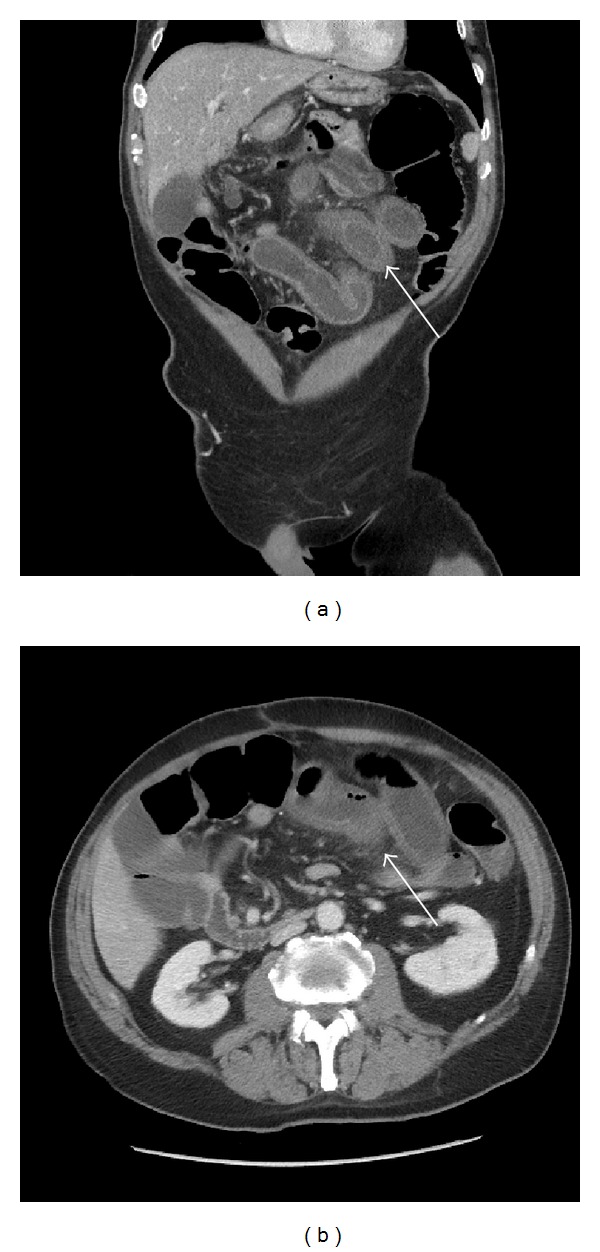
(a) A coronal reformatted image of the CT abdomen and pelvis demonstrating multiple dilated, fluid filled small bowel loops, with thickened and enhancing mucosa in keeping with bowel wall oedema (*Arrow*). (b) Axial images again showing thickened oedematous small bowel loops with surrounding inflammatory fat standing (*Arrow*).

He was started on oral capecitabine, with his first cycle consisting of 1000 mg/m^2^ BD for 2 weeks (a 20% dose reduction in order to identify any early toxicity). This cycle was completed without toxicity other than a mild facial skin rash and the second cycle dose was increased to 100% (1250 mg/m^2^ BD). On day 16 of the second cycle our patient complained of reduced appetite, lower abdominal discomfort, diarrhoea (grade 3), and giddiness associated with falls at home. He was admitted and found to be hypotensive, dehydrated, and pyrexial with a WCC of 5.3 (Neutrophils 3.2) and CRP of 141. He was rehydrated and started on tazocin, gentamycin, and metronidazole. Over the next two days he developed marked abdominal distension and his diarrhoea persisted in spite of Imodium. Abdominal X-ray demonstrated distended loops of small bowel. CT Abdomen illustrated a patent ileocolic anastomosis with a non-distended large bowel and no evidence of mechanical obstruction. There were fluid distended loops of small bowel with abnormal thickening and inflammatory changes of the wall of the distal loops of ileum. The radiological findings were suggestive of an acute ileitis (Figure 1).

By day 3, blood, urine, and stool cultures remained negative. Stool sample also tested negative for occult blood and parasites. Actinomycosis and Yersinia serology were negative. Antibiotics were switched to meropenem and he was provided with parenteral nutrition. He received 2 weeks of conservative management during which his symptoms greatly improved before being discharged home. The patient had no further chemotherapy and remains well 9 months postoperatively with bowel function restored to normal. 

## 3. Discussion

Colon Cancer remains one of the most common cancers worldwide and a leading cause of death in the western world [1]. Surgery remains the mainstay of treatment, however, postoperative adjuvant chemotherapy reduces the mortality rate by 33% among patients with stage III colon cancer. A meta-analysis concluded that patients with stage II disease could also benefit, but to a lesser extent; the absolute benefit being only 50% that of patients with stage III disease [2]. Clinical and biological prognostic factors need to be taken into account when discussing adjuvant chemotherapy with stage II patients and in this case the patient had several poor prognostic factors (emergency presentation, tumour perforation, and MSS), which favoured the use of adjuvant chemotherapy [2].

Two of many treatment options available to patients include a weekly bolus 5-FU or capecitabine. Capecitabine is an oral fluoropyrimidine which is preferentially converted to fluorouracil in tumour tissue, by the way of a three-step enzymatic cascade. The final stage of conversion to fluorouracil is catalysed by thymidine phosphorylase, which is appreciably more active in tumour than in healthy tissue [3]. The X-ACT trial, which compared the use of capecitabine with the MAYO regimen of bolus 5-FU/LV in patients with stage III colon cancer demonstrated similar efficacy but with reduced grade 3/4 toxicity [4]. It showed that patients prefer an orally administered treatment and the trial also demonstrated reduced health economic costs. The side effects of capecitabine include fatigue, nausea, vomiting, diarrhoea, palmar-plantar syndrome (hand-foot syndrome), anorexia, and cardiotoxicity [5].

Our patient displayed marked gastrointestinal toxicity following his increased dose of oral capecitabine. The CT finding of acute ileitis was corroborated by two specialist gastrointestinal radiologists. Once his clinical picture had improved a repeat CT scan showed a similar marked improvement. White cell and neutrophil count remained within normal range for the duration of his admission.

To date, only 7 cases of small bowel toxicity through the use of intravenously administered 5-Fluorouracil have been documented in the literature [6]. In all instances, although clinical findings varied amongst patients, all developed acute abdominal pain and diarrhoea. One possible explanation for this observed toxicity is that 5-FU can significantly decrease gastric mucosal blood flow [7].A report from the Netherlands recently described one case of a patient with metastatic rectal cancer who suffered similar toxic effects following the use of oxaliplatin, bevacizumab, and capecitabine [8]. To date, Roche Drug Safety identifies the incidence of suspected ileitis to be less than 0.01% of their patient population. As we have documented above and also what has been illustrated in other cases, the management of such patients is mainly supportive. The discontinuation of the chemotherapeutic agent, followed by a period of parenteral nutrition, rehydration, and empirical antibiotics allows for adequate bowel rest and the restoration of normal function [6, 8]. 

## 4. Conclusion

This is the first suspected case of ileitis secondary to capecitabine use in the English literature. The efficacy of capecitabine as adjuvant therapy in colon cancer is well demonstrated and its lower toxicity rates when compared with 5-FU makes it an increasingly more favourable option for patients. This case highlights the awareness of a potentially severe side effect related to the direct use of capecitabine, yet through the early identification of symptoms patients can be managed conservatively.
